# Caring for older adults during the COVID pandemic and beyond: experience from a specialized tertiary facility for the care of older persons in a low resource setting

**DOI:** 10.11604/pamj.supp.2020.35.2.24521

**Published:** 2020-07-02

**Authors:** Lawrence Adekunle Adebusoye, Eniola Olubukola Cadmus, Elizabeth Oluwatomi Labaeka, Samuel Ayosina Ajayi, Olufemi Oluwole Olowookere, Jesse Abiodun Otegbayo

**Affiliations:** 1 Chief Tony Anenih Geriatric Center, University College Hospital, Ibadan; 2 Department of Community Medicine, College of Medicine, University of Ibadan, Oyo State, Nigeria; 3 Department of Medicine, University College Hospital, Ibadan, Oyo State, Nigeria

**Keywords:** COVID 19 pandemic, care of older persons, Care in Place, facility-based, Nigeria

## Abstract

The ongoing Coronavirus disease (COVID-19) pandemic has markedly changed health care provisions and arrangements for patient care. Older adults are most susceptible to worse outcomes. The public health impact of the disease in terms of morbidity and mortality has necessitated the evolution of management protocols for effective care of older persons. This review describes our experience during this period attending to the healthcare needs of both the acutely ill and clinically stable patients at the first purpose-built facility for the care of older persons in Nigeria, the Chief Tony Anenih Geriatric Centre (CTAGC), University College Hospital, Ibadan. A major strategy recommended by the World Health Organization was a lockdown with restricted movements and laid down rules for engagement. As such, the CTAGC also embarked on steps to ensure patient safety as well as effective care. Prior to the lockdown, targeted activities included fumigation of the centre as well as health education and promotional activities. Measures were put in place to care for up to 95% of our patients at home. Thus, a “Care in Place” approach was adopted to enable them to take ownership of their care. Ambulatory older patients were seen on an out-patient basis following scheduled appointments after a telephone consultation through the hospital’s designated lines. Clients were managed for their routine health conditions which were mostly non-communicable diseases (NCDs). Also, acutely ill older patients were admitted for acute exacerbation and/or complications of their chronic morbidities. Importantly, 60% of admitted patients presented with COVID like symptoms but they all tested negative for COVID 19. Based on our experience at the CTAGC, older persons can be successfully managed through a “Care in place” approach in a resource-poor setting during pandemics with high infectivity rates such as COVID 19. The information hereby generated is beneficial for future practice.

## Perspectives

The 2019 Coronavirus (COVID-19) disease pandemic is a global reality and has led to changes in health care provisions and arrangements. The disease is caused by the Severe Acute Respiratory Syndrome Coronavirus 2 (SARS-CoV-2) and was first reported in Wuhan Province of China [1]. The SARS-CoV-2 virus has a predilection for affecting the respiratory tract and up to 67.8% of patients managed for COVID-19 disease in municipal areas of China had a cough on admission with radiological evidence of chest affectation in 54.6% of patients [2]. In countries hardest hit, older persons have been documented to have a higher risk and constituted the higher mortality [3]. However, there is a paucity of information regarding the level of affectation of older adults in low- and middle-income countries (LMIC) such as Nigeria. Considering the high risk of older persons to the infection based on the inherent vulnerabilities such as underlying morbidity most of the scare among older persons is well-founded. Older persons are more prone to infections than younger adults for various reasons. Some of these factors are associated with the physiological changes that occur as a result of ageing while other factors are either acquired or genetically based [4]. Immunosenescence is one of the changes that occur in older persons. This change occurs as a result of an increase in the pro-inflammatory and anti-inflammatory cytokines which cause a dysregulation of the innate immune response to infection [5]. The resulting age-related chronic inflammation contributes to the dysregulation of the innate immune responses. Also, the B- and T-cell functions in the adaptive immune system are both diminished [5]. Immunosenescence and the accompanying inflammatory state have been implicated in the occurrence of increased incidence of pulmonary infection in older persons [6]. Considering this underlying increased vulnerability to pulmonary infections, it is, therefore, important to protect older persons from exposure to the virus. This approach is critical especially as respiratory tract infection is notably a common cause of morbidity and mortality in older patients [6].

The predisposition of older persons to the COVID-19 disease may be further explained by the presence of non-communicable diseases (NCDs) which account for about 87% of the disease burden in both high-income countries (HIC) as well as LMIC [7]. The burden of NCDs in many LMIC is projected to increase by a range of more than one-half in low-income countries and more than three-fourths in middle-income countries by the year 2030 [7]. The double burden of infectious and NCDs further compounds vulnerability of older persons to the disease. Also, due to the more stringent screening conditions in place, the ongoing COVID-19 pandemic has further made accessing health care difficult for older adults. In addition to the challenges faced, there is increased prolongation of hospital visits. Furthermore, there are reports that underlying infrastructural and infection control deficits in many LMIC further contribute to the difficulty in the hospital management of older patients during the COVID-19 pandemic. For instance, Garg *et al*. [8] in India reported limited physical space and queuing capacity in the health facilities. Also, inadequate exit and entry points, poor ventilation, inadequate facilities for handwashing and hand hygiene were highlighted. The situation in Nigeria is not far from this reality as the design of many general clinics that are not purpose-built for the management of respiratory infections is not well suited for the limitation of the spread of airborne or droplet transmissible diseases. These deficiencies emphasize the importance of targeted measures to reduce the need for hospital visits for older persons during the ongoing pandemic. These measures include the provision of alternative consultation means for the patients with the benefit of reduced length of stay for those who have to visit the hospital and ultimately reducing the risk of exposure to the virus.

Among the measures adopted to ensure older persons are kept away from the hospital as much as possible thereby limiting exposure to the virus is the adoption of self-management practices. Self-management is defined as: ‘routine tasks undertaken by an individual to properly control or reduce the impact of a disease on their well-being’ [9]. Although self-management is beneficial, the concept is underutilized in the management of older patients with chronic conditions [10]. Several factors notably enhance the self-care ability of individuals. These include their experience, knowledge of disease condition, available training as well as self-determination. Other factors include the individual´s responsiveness, perceived responsibility, engagement and adaptation [10]. During the COVID pandemic, access of older persons to health facilities was further limited as many facilities were either shut down or the older persons were worried about contracting the infection and so they preferred to stay home. Therefore, the ability to self-manage and report clinical conditions to physicians via the use of technology was also a welcome option.

The use of telemedicine is not alien in many spheres and is effective in the monitoring of older patients with multimorbidity while enhancing their optimal care [11]. However, the utilization of telemedicine in Nigeria is fraught with challenges militating against its successful implementation. These include an erratic electrical supply, as well as the lack of qualified manpower to operate and maintain the units [12]. Due to these limitations the use of Global Systems for Mobile Communication (GSM) was introduced as a cost-effective model in the practice of telemedicine with the introduction of the Mobile Doctor Concept (MDC). Initially, the concept enabled clients to speak with a medical doctor or other qualified health personnel at a rate of 100 Naira (about 68 cents) per minute [12]. Although the rate was rather expensive, GSM call services have become more affordable over the years with rate reductions as low as 24 Naira (about 5 cents) per minute. This rebate made the use of the technology feasible during the COVID-19 era because it did not warrant any capital or resource investment from an already strained health sector. Before the COVID-19 pandemic, telemedicine was not a popular means of consultation in Nigeria but has been brought to the fore now. Furthermore, care of older persons is evolving in many LMIC such as Nigeria. As a whole, most healthcare centres across the country have suboptimal facilities and are ill-prepared for the ongoing pandemic. Furthermore, there are few specialized care facilities for older persons as well as long term care (LTC) options. Also, available facilities are mostly sub-standard and poorly regulated in comparison with the high-income countries (HIC). Other challenges faced by older Nigerians are low resources further complicated by early retirement, unpaid pensions, and the high unemployment rate among the younger population. This makes it difficult for the children to adequately provide for the needs of their older relatives.

**Our experience:** this review describes our experience in the provision of care for older persons at the first purpose-built facility for older persons in Nigeria, the Chief Tony Anenih Geriatric Centre (CTAGC). The Centre was established in 2012 and over the years, there has been a steady increase in the number of patients from the first six months to date. By the end of April 2020, a total of 18,496 patients had been enrolled. There are nine service areas at the CTAGC tailored to the needs of older patients. These are outpatient, in-patient, physiotherapy, dietetics and medical social work. Other specialities include surgery, dental, rehabilitative, health and safety service areas. The daily turn-out is between 110 and 140 older patients. Services at the centre are at 50% of what obtains in other parts of the hospital. The CTAGC uses the Electronic Health Record (EHR) system which was achieved through the All-Purpose Medical Information System (APMIS). The transfer of medical information of patients from registration point to all other units in the centre is carried out electronically. Each service point of the centre is provided with laptops supplied by the Local Access Network (LAN) and other wireless networks. When necessary, patients are reminded of their clinic appointment a day before the consultation day by Short Message Service (SMS). This has reduced the patient-waiting time, improved the overall patient-satisfaction and greatly encouraged the patients. Health education is given daily to provide patients and their carers with much needed and relevant information on personal hygiene, nutrition, health maintenance, exercises and appropriate medication use.

### Routine outpatient and inpatient service provision

***The process of receiving care at the outpatient unit:*** this all-inclusive facility allows older patients to have easy access to health care services through a smooth flow of activities within the same hall. Older persons are ushered into the clinic by hospital assistants who are stationed at the entrance of the clinic. The assistants help the patients alight from the vehicles and offer wheelchairs to patients who are not ambulant. This gesture has received commendations from the patients and their relations who had hitherto not been exposed to such. Patients are registered in the health record station from where they move to the payment-point, then to the nurses´ station where their vital signs are measured. Thereafter, patients are then directed to the consulting rooms to see the doctors. After the clinical consultation, they proceed to the pharmacy unit for their drugs, while those who require laboratory investigations are attended to by the scientist in the laboratory room. Other investigations provided at the Centre include the measurement of bone mineral density which has proven beneficial in the detection and management of osteoporosis. This is assessed using the Dual Emission X-ray Absorptiometry (DEXA) machine at no cost to the patient. Likewise, older patients in need of investigations for cardiac abnormalities are investigated Electrocardiogram (ECG). As with other services, the cost of the ECG at CTAGC is at 50% of what is being paid elsewhere in the hospital. Health education is given daily to the patients and their carers on personal hygiene, nutrition, health maintenance, exercises and appropriate medication use by all the constituent units. Acutely-ill elderly patients are treated as emergency cases at the centre. Those needing hospitalization are admitted into the wards. Their management is facilitated by equipment such as the crash carts, pulse oximeters, glucometers and electrocardiogram. Training and retraining of the medical personnel at the centre in Basic Life Support (BLS) and Advanced Cardiac Life Support (ACLS) courses are carried out routinely.

**Pre COVID-19 preparedness:** prior to the COVID pandemic, older patients at the geriatric centre were routinely given health talks by public health nurses. These include measures for healthy lifestyles such as good dietary habits, increased physical activities, alcohol use moderation and weight modification interventions. Other topics include avoidance of injuries, judicious use of medications, tobacco cessation and fall prevention measures. Part of the care plan of the patients at the Centre include education of patients about their clinical conditions including identifying triggers and red flags where applicable. Further measures were immunoprophylaxis with the pneumococcal vaccine using the Centre for Disease Control and prevention (CDC) guideline [13]. Chemoprophylaxis with daily aspirin as well as dietary supplements such as Vitamin B, ascorbic acid and Calcium were administered based on patients´ requirement. The Medical Social Work Unit (MSWU) routinely tracks older patients through the use of SMS to remind them of their clinic appointments. Those who default on clinic appointments are tracked by phone calls and home visits. The ward arrangement for admissions is segregated to a maximum of two patients per room to limit hospital-acquired infections.

### The COVID era

**Pre lockdown:** the Federal Government of Nigeria through the directive of Presidential Task Force on COVID-19 announced that the country would undergo a lockdown by 11 pm on Monday 30th March 2020. This directive necessitated the restructuring of existing facilities at the Centre. Prior to the lockdown, instructions were given to the patients with emphasis placed on health promotional activities. Patients were advised to stay at home, stay safe and limit visitations both within and outside their homes including visits by family members. This was challenging as the African tradition encourages the extended family system where families are closely knit and may even live together [14]. As such many older persons were introduced to the use of digital technology and social media as a communication bridge. Also, in Nigeria, there is a high level of religiosity and faith-based activities which keeps older people occupied. According to the World Values Survey between 2010-2014, 94.3% of older adults in Nigeria described themselves as religious [15]. This propensity made the recommendations that older persons should avoid religious places and other social gatherings difficult. Nevertheless, the older clients were advised to remain physically active at home and carry out indoor exercises. Further, they were admonished to listen to the daily media briefings of the Presidential Task Force of the Federal Government of Nigeria on COVID 19. Likewise, the need for strict medication compliance was emphasized as well as the benefits of adequate nutrition. Besides, due to the transition from dry season to the rainy season, the clients were advised to ensure they take fluid of at least three litres per day to maintain hydration. Lastly, the MSWU was re-energized to intensify effort on tracking and were mobilized for the task ahead.

**During the lockdown period:** in terms of preparation, there was fumigation and wash down of the entire geriatric centre. Thereafter, there was a meeting with the hospital´s (University College Hospital) COVID-19 taskforce, which had in attendance all heads of units in the centre, doctors and administrators. Following the meeting, a decision was made to close down service units which warranted close contact between doctor to patient and patient to patient These included the dental unit, eye clinic and subspecialty units such as the healthy ageing, rheumatology, endocrine and neurology clinics. Next, a “Care in Place” policy was implemented which involved the provision of care for otherwise ambulatory patients within the comforts of their homes to avoid unnecessary hospital visits and risk of infection to the virus. Our goal was to have at least 95% of our older patients care for themselves at home with telemedicine support. Considering that the pre-COVID patient load at the clinic was 110-140 patients a day, to limit crowding, a maximum of 6-7 older patients per day were expected to be present at the Center during the lockdown period. As such the plan was to utilize electronic consultation to address their health issues by giving out phone numbers of the doctor-on-call, nurse-on-duty and the Head of the centre.

A previous study among older patients attending the CTAGC revealed that the common morbidities were hypertension (45%), diabetes mellitus (10.4%), and musculoskeletal degenerative diseases -spondylosis and osteoarthritis (13%) [16]. Based on this finding the team was guided on the necessary information the clients needed to be able to manage their health conditions at home. The older patients were taught how to manage their hypertension at home. This included regular weekly measurement and recording of the blood pressure using the sphygmomanometer. Likewise, diabetic patients were counselled on self-management of their conditions by monitoring their blood glucose using a glucometer and recognition of hypo- and hyper-glycaemic symptoms. On the other hand, patients with pulmonary problems such as Chronic obstructive Pulmonary Disease (COPD) were taught how to use their inhalers and diskus. All patients were expected to document the readings from their devices in a diary to facilitate electronic consultations when needed or clinic visits.

### Hospital care during the lockdown

***Outpatient care:*** an open shelter was provided for the visiting ambulatory patient as previously stated, the expectation was that no more than five percent of the normal patient load would present each day. Safe distancing (2 metres) was maintained in the sitting arrangement. The use of facemasks was made compulsory for the older patients and the accompanying carer(s). Thereafter, health education by public health nurses on health and safety as well as home care was given to them. From the holding bay, older patients were called one after the other. When a patient is called, the patient and the carer had mandatory hand washing and the use of a sanitizer before entering the Out-Patients Department (OPD) under the supervision of a designated health attendant. A maximum of one carer was allowed to accompany a patient. At the clinic the health care workers-maintained safety precautions and personal hygiene. The sitting arrangement in the consulting rooms was such that ensured adequate physical distance between the doctor and the patient during the consultation; The doctor sat at the table and the patient’s seat was moved about 2 meters away close to the window. All windows in the consulting room were left open during the consultation. Close contact with patients was limited to only a brief focused examination. This is a contrast to the consultation sitting arrangement in the Pre COVID era where both the patient and the doctor were seated at an arm´s length from each other.

***Inpatient care:*** throughout the three-month lockdown period, a total of 10 older patients with equal sex distribution were admitted. The diagnoses on admission constituted both infectious diseases and NCDs. Information from some health care facilities showed that older patients with COVID-like symptoms were refused treatment on the assumption that they were infected which contributed to the mortality and stigmatization of the older persons. However, at the Center, treatment was given to all the older patients that presented at the Centre. Clinical diagnosis of patients who were managed on an in-patient basis included anaemia secondary to pancreatic cancer, pulmonary thromboembolism, congestive cardiac failure and malaria. Other conditions included gastritis, chronic diarrhoea with electrolyte imbalance, bleeding cancer of the prostate, lobar pneumonia, pericardial effusion secondary to a tumour and a hyperglycemic state.

Interestingly, six out of the ten patients (60%) admitted presented with COVID-19 like symptoms such as respiratory distress, fever, cough and generalized body weakness out of whom five out of 6 (83%) had the trio of fever, cough and breathlessness. One patient had a combination of fever and body weakness. However, all the patients were tested for the SARS-CoV-2 virus while on admission but were negative. The laid down protocol before presentation in the hospital necessitated that patients called any of the previously designated phone lines ensured readiness to receive acutely ill older patients. The vehicle conveying the ill patient was allowed into the facility. A doctor dressed in personal protective equipment (PPE) assessed the patient and admitted the patient. The goals of admission were to immediately stabilize the patient and promptly manage the acute illness the patient presented with. Lastly, the target was early discharge as soon as the patient was deemed fit to continue recuperation and care at home. Each patient was limited to one designated carer that was allowed inside the ward. This was difficult because relatives are critical stakeholders and participate actively in the management of older patients in the traditional African setting. Other COVID-19 safety measures including mandatory hand washing, regular sanitation, the use of appropriate PPEs was continued. Sadly, there was one mortality but this was due to an acute exacerbation of a chronic condition.

## Conclusion

The COVID-19 pandemic has brought to fore the importance of empowering patients to be actively informed about their clinical condition and to safely carry out self-management. Telemedicine can be a safe alternative where physical consultation is constrained.

***COVID-19 and beyond:*** there is a realization that the COVID-19 pandemic will be with us for some time to come. Therefore, there is a need to put in place practice for the future. Although the projected avalanche of mortality estimated in many LMIC such as Nigeria did not materialize, targeted action is essential to ensure older patients stay safe while in our care and their homes. Likewise, there is a need to further engage the “Care in Place” approach as a simple, cost beneficial and highly effective tool. The experience at the CTAGC has shown that it is possible to care for older persons through their involvement and within their homes in resource-poor settings if health education, adequate preparations and sensible measures are put in place. Limited resources should not be an excuse for a lack of innovative solutions for the situation.

**Lessons learned:** 1) During a highly infectious pandemic, the majority of older patients can safely be managed through a “Care in Place” approach 2) Although older persons are vulnerable to COVID-19 infection, presentations to the hospital during such pandemics should be limited to those with complications and acute exacerbation of their chronic diseases. 3) Some clinical presentations will mimic the symptoms and signs of COVID-19 infection, for example, pulmonary thromboembolism, community-acquired pneumonia and even malaria. Health education with an emphasis on the health and safety of health care workers and older patients is paramount.

**Recommendations:** preparedness is key. The EBOLA epidemic (2014) in Nigeria documented the success of having a laid down protocol. Thus, there should be Pre-infection preparedness to combat health situations such as the COVID-19 pandemic. This should be at three strategic levels of intervention. 1) Individuals should be equipped with adequate information and health education. Through the observance of rules, individuals must be enabled to take personal responsibility to protect themselves and others. 2) Healthcare facility: Infrastructure strengthening (PPE, Mask), Innovation and developments of laid down Standards of Operating Procedures (SOP), and training and retraining of staff. 3) Government: Establishment of specialized facilities for the care of older persons, and adequate funding of the health sector.

## Competing interests

The authors declare no competing interests
